# The Need for Peer Support and Codesigned Services: A Qualitative Study to Understand Diabetes Education Program Needs of Adolescents With Type 1 Diabetes

**DOI:** 10.1155/pedi/1843544

**Published:** 2025-06-24

**Authors:** Ashley H. Ng, Wenbo Peng, Giuliana Murfet, Marlene Payk, Siobhan Barlow, Shanshan Lin

**Affiliations:** ^1^School of Public Health, Faculty of Health, University of Technology Sydney, Sydney, Australia; ^2^Monash Centre for Health Research and Implementation, Monash University, Melbourne, Australia; ^3^Diabetes Centre, Tasmanian Health Service – North West, Burnie, Australia; ^4^School of Nursing, College of Health and Medicine, University of Tasmania, Hobart, Australia; ^5^Type 1 Foundation, Victoria, Australia

**Keywords:** adolescents, diabetes education, diabetes/type 1 diabetes, self-management, social support/gamification

## Abstract

**Background:** Developmental challenges of adolescence, such as puberty and social pressures, exacerbate the complexity of managing type 1 diabetes (T1D) as they transition from pediatric to adult care. However, there is a paucity of codesigned, evidence-based diabetes education and support programs and services to guide adolescents through this transition.

**Objective:** This study aimed to explore the experiences, perspectives, facilitators, and barriers faced by adolescents with T1D in diabetes education and program services and to identify feasible approaches to support them as they transition from pediatric to adult care.

**Methods:** Semistructured interviews were conducted with 13 adolescents aged 13–19 years with T1D. Thematic analysis was used to understand participants' past experiences, facilitators, barriers, and preferences regarding diabetes education programs and services.

**Results:** Participants highly valued the opportunity to meet with peers living with T1D and the emotional support from those interactions. Participants also highlighted the need for age-appropriate content and interactive learning experiences. Suggested gamification features were well-received, with participants emphasizing the importance of interactivity. While there was not a strong preference between virtual or in-person game formats, it was suggested that online options offered flexibility and inclusiveness regardless of physical abilities. Participants were not as enthusiastic for a one-on-one live chat compared to an online community chat, again, for the opportunity for peer support.

**Conclusion:** The study highlights the value that adolescents with T1D place on peer support that arises from opportunities to meet others through in-person events. It was evident that codesigning diabetes education programs and services with adolescents with T1D is key to develop tailored offerings for this population.

**Summary**

• Adolescence is an optimal time to introduce diabetes education to support the independence of self-management, while adolescents are still highly engaged with clinical services.
• Interactive, face-to-face opportunities for diabetes education and peer support in an environment they are comfortable with are preferred by adolescents compared to online learning modes.
• Learning content and style needs to be age appropriate to ensure it meets the needs of the target audience.

## 1. Introduction

Managing type 1 diabetes (T1D) in adolescents requires careful consideration due to unique psychological, physical and social needs, which can significantly impact the future management of T1D [1]. Diabetes self-management requires daily glucose monitoring, insulin administration, personalized nutrition, and physical activity, which become challenging for adolescents balancing competing priorities including studies, social life, and paid employment [2]. Notably, the onset of puberty drastically increases the risk of vascular complications in adolescents with T1D [3]. In parallel, evidence suggests that increasing independence in health management leads to disconnection from the pediatric healthcare team [4], with T1D adolescents often experiencing anxiety and diabetes burnout, further delaying the effective person-centered transition to adult care [5].

Despite challenges faced by adolescents with T1D, few evidence-based resources are available, with most research focusing on the transition from pediatric to adult care rather than solutions for this developmental life stage [6]. Transitioning emphasizes an increase in independence, assumption of responsibility and self-management tasks; in contrast, pediatric diabetes care takes on a family-focused approach, with the main caregiver managing the condition in conjunction with a multidisciplinary diabetes team [7]. Research suggests that adolescents rely on healthcare professionals for gradual guidance through this transition to adult care, with parents playing a supportive role [6].

While healthcare professionals recognize the value, acceptability, and feasibility of effectively implemented and sustainable models of care, there is a lack of transition models that support person-centered care [5, 8]. Current evidence has primarily focused on exploring the attitudes and experiences of adolescents using standard transition models of care, which are important to assess effective care [7]. However, the unique needs of adolescents are rarely addressed in these models of care [8]. Inadequately developed transition care can lead to preventable comorbidities, additional long-term healthcare costs, and poorer quality of life with lifelong distress [9].

The current study aimed to understand the health concerns faced by Australian adolescents with T1D and identify ways to address those needs effectively. Adolescents in this study have been defined as between the ages of 13 and 19 years, in accordance with the World Health Organization [10]. The specific objectives were:

1. To explore adolescents' experiences and perspectives, including barriers and facilitators to diabetes education and program services.
2. To determine the features and functions of a diabetes education program to address the needs of adolescents for diabetes self-management.

## 2. Methods

This study used a phenomenological qualitative approach to explore the experiences, facilitators, barriers and needs of adolescents with T1D [11]. Adolescents aged 13–19 years old with T1D were invited to take part in a 45–60 min semistructured interview to better understand their experiences, perspectives, and diabetes education needs. Recruitment was conducted through various channels with flyers distributed across diabetes consumer organizations, diabetes clinics in urban and rural Australia and relevant social media channels with an aim for geographical representation across Australia. Recruitment was ceased when geographical representation and data saturation were achieved, defined as no additional themes being identified [12]. Eligibility criteria included meeting the age range and diagnosis criteria, residing in Australia and be able to communicate in English. Verbal consent was obtained from all participants, including parents or carers for those under the age of 18 years, prior to commencing the interview. For participants under the age of 18 years, additional informed consent was required from parents or carers, who were invited to be present during interviews. Ethics approval was obtained from the University of Technology Sydney Medical Research Ethics Committee (ETH23-8213).

A semistructured interview guide was designed using an inductive logic of inquiry on perceived needs in diabetes education, previous experience of diabetes education programs and services and an ideal program or service with prompts to digital health interventions and features. Questions were developed in conjunction with the project team, which included expertise from a lived experience academic (AN), a parent of a child with T1D (SB) and healthcare professionals specializing in diabetes (Credentialled Diabetes Educators, Advanced Accredited Practising Dietitians and Nurse Practitioners) who work with youth and those in transition (SL, MP, and GM), and used for all interviews (Supporting Information 1: Appendix 1). Interviews were conducted by an experienced qualitative researcher (AN) with diabetes lived experience, who had no prior relationship with participants. The interviews were conducted either by phone or videoconference via Zoom and audio recorded for transcription purposes. As part of the member checking process, transcripts were returned to participants to ensure accurate information was captured. Upon completion of the interview, participants were provided with an AUD50 gift card.

Data analysis employed thematic analysis in the form of template analysis, as described by Brooks et al. [13]. This approach involved first defining *a priori* themes after familiarization with the data after three transcripts to develop a template (AN). The coding template was then reviewed and confirmed by members of the team with clinical experience as a Credentialled Diabetes Educator (SL) and expertise in qualitative analysis (WP) to ensure interpretation of the data within an appropriate context and to mitigate confirmation bias. Subsequently, the developed coding template was applied to the entire dataset, with themes being modified or added as necessary during the coding process.

## 3. Results

### 3.1. Demographics

Of the 43 expressions of interest recorded during the recruitment phase, 13 adolescents with T1D met the inclusion criteria and completed the interviews. Of these, 54% (*n* = 7) were males, 54% (*n* = 7) resided in Victoria, while the other participants were from Queensland (*n* = 3), South Australia (*n* = 1), New South Wales (*n* = 1), and Tasmania (*n* = 1). The average age of participants was 15 years (range 14–18 years) and the average duration of diabetes was 4 years (range 1–14 years).

The majority of participants reported using a continuous glucose monitoring system with an insulin pump, with only one using multiple daily injections. All participants reported regular in-person visits to an endocrinologist or pediatrician (either every 3 or 6 months), with 10 occasionally seeing either a Credentialled Diabetes Educator and/or an Accredited Practising Dietitian. Two participants reported regular visits to a psychologist. Most participants indicated that they mostly self-managed their diabetes with occasional input from their parents, often the mother.

### 3.2. Awareness of Diabetes Camps and Events

All participants reported to have attended at least one diabetes camp or event, such as social meetups with other families, webinars or online conferences. In almost all cases, parents were the ones who discovered these events and encouraged participation. Participants were often unaware of these events as they reported that the information was typically targeted at parents. One participant expressed a preference for learning about these events from their peers rather than being instructed by an adult to attend.

*“it's more just getting the awareness out there because sometimes it's hard to know if these things are going on unless you're searching for it” – participant 010*

### 3.3. Value of Diabetes Camps and Events

#### 3.3.1. Emotional Support

Participants who had attended a diabetes camp or event, had overwhelmingly positive feedback. Participants reported feeling emotionally supported as they met peers who could relate to their experiences of living with diabetes. Many reported that hearing about others' experiences and struggles made them feel less isolated. For some, these events led to ongoing friendships and a support network related to diabetes.

*“*[*diabetes camp*] *helped me a lot just knowing that it's not just me. And it's just nice knowing that everybody else is struggling with the same thing I struggle with” – participant 005*

*“I think building those connections with other people who are in the same situation as you is very important and mentally it helps a lot” – participant 018*

*“…a lot of my good diabetes friends help me a lot. Like if I ever get sick of it and need to just talk to them*, *they're really good to talk to*, *just because they understand” – participant 010*

#### 3.3.2. Accessing Information in Age-Appropriate Formats

Participants highly valued diabetes-related information shared in an age-appropriate format as many written resources and events were pitched for a younger age group or adults. Events, such as camps provided the opportunity for information to be shared in an age-appropriate manner. Participants reported that discussions at camp raised questions or topics they had not considered, prompting them to think about different aspects of diabetes management. Ensuring that the content and format of these camps and events were age-appropriate was critical for encouraging future participation.

*“I feel like there should be more events around*, *for*, *like adolescents and older kids cause a lot of the events are either for younger kids or like adults. There's not a lot of in between ones” – participant 009*

#### 3.3.3. Helping Friends and Family Understand Diabetes

Participants reported that the ability to bring family and friends to diabetes events was important. Participants emphasized the importance of their family and friends understanding how to support them, particularly during critical situations, such as a severe hypoglycemic episode. Additionally, participants reported the importance of hearing credible diabetes information to dispel diabetes misinformation. Together with understanding the basics of diabetes management, majority of participants perceived that their family and friends needed to hear from others what it is like to live with diabetes to understand the broader emotional burden that the condition brings. Some participants shared their perception that parents often felt they understood what it was like to live with diabetes as they played an active role in their child's diabetes care. A few participants advocated for having diabetes events where parents had an opportunity to connect in their own space to share their experiences and learnings.

*“my parents think that they know what it's like for me to live with diabetes*, *but they actually have no clue like I got into an argument with my mum once. And she was like*, *I know exactly what it's like to live with diabetes. I live with you*, *and I was like*, *you live with me. You don't live me” – participant 011*

*“I prefer to just send my parents to a session without me. Cause I feel like I have lived it. I know it all. They don't actually really know much*, *and I don't think I need to sit through…it might be worth for them…to just understand what it's like to actually live with*, *like the kind of mental toll and the like exhaustion…” – participant 015*

*“it's like also a good place where parents can connect with other parents*, *can talk about their child and what they've experienced*, *and just also get advice from people who have more experience with Type 1 and families that have more experience with Type 1” – participant 018*

### 3.4. Accessing Diabetes-Related Information

A few participants reported using social media to gain more information about diabetes. Through social media, hearing stories from others living with diabetes provided valuable insights into managing their own diabetes and learning different strategies or “hacks”. For the other participants, information about diabetes-related camps, events and general information was mostly conveyed by their diabetes clinicians and parents. Consequently, participants suggested that parents often played an active role in diabetes groups on social media platforms or sought out diabetes organizations for credible information.

*“for a long time I struggled with accepting the fact that I like struggled with this*, *and no one else did. Because in my community I'm like one of the only people that has diabetes. But over the past*, *like 2 years I've accepted that and started following like a bunch of new people and like there's this one account…she posts like so much about new technology*, *and like how she goes day to day like traveling in with diabetes and stuff. And I find her content like*, *really helpful” – participant 011*

Participants were also invited to indicate topics of particular interest, which they wished to receive more information about. These topics, ranked by frequency of mention, are presented in Table 1.

### 3.5. The Ideal Diabetes Education Service

#### 3.5.1. Modality of Program Delivery

All participants expressed a preference for in-person diabetes education programs over virtual or hybrid options. Reasons included the ability to be present and focus on the information, ease in making personable connections, and more effective interaction with peers and presenting health professionals. Participants desired interactive content delivery rather than didactic presentations.

*“it's when you can really like interact with people more and …you feel like you're more in the moment…I just think*, *yeah*, *probably face-to-face I think is a way I'd enjoy it more” – participant 014*

#### 3.5.2. Timing of Program

Responses varied regarding the ideal timing for a diabetes education program. Preferences included weekends, school holidays or a combination of both due to extracurricular activities like sports, or part-time or casual work or access to transportation support (e.g., access to someone available to drive participants to the event, particularly if family and friends were invited to attend). Participants acknowledged that there was no ideal time, but with enough notice, they could plan around the event and reschedule other conflicts if needed.

#### 3.5.3. Gamification

While participants preferred in-person events, almost half opted for a virtual video game when presented with a diabetes educational game or in person physical game options. Reasons included the flexibility to complete the game at their own pace, the ability to save progress, and inclusivity regardless of physical ability. Participants who had a stronger link to sports chose a physical game rather than a virtual offering.

*“*[*A*] *video game is probably a lot more easier. You can just go on and do it whenever you want*, *and you don't have to have a certain amount of time set aside for it.” – participant 003*

Participants emphasized the importance of age-appropriate, interactive game formats. Many participants preferred problem-solving or adventure type genres of games, providing examples, such as counting the number of carbohydrates in a meal before the timer runs out to unlock the next clue or selecting the correct response to advance to the next stage of the game.

*“it's not just like passive…I'm not just reading information*, *because I probably won't take it in…but if I have to actually click the right option or something…” – participant 015*

*“So something like*, *guess the number of carbs in 30 s to unlock this thing or something…Puzzles then get you thinking and then the adventure makes you want to continue playing for the story as well.” – participant 014*

All participants agreed that incorporating a reward or scoring points to redeem prizes throughout the game added a fun, competitive edge and would increase motivation to complete the education components or modules. While the ability to earn points serves as a good motivator for some to work towards, some participants expressed importance in incorporating other ways to recognize their efforts. Particularly if individuals were struggling with the program, the inability to score points had the potential to make them feel worse about themselves, diminishing motivation for learning and progress. One participant suggested the option of having a goal to work towards, such as finishing the storyline of the game, as a good motivator.

*“I think that it would be motivating if you're doing good. But I also think that like if I was having a really rough time with my diabetes*, *and I didn't like get any points or something*, *it would just make me feel even worse” – participant 011*

When asked about the type of reward they would prefer, most participants were ambivalent with the options provided, such as practical diabetes accessories, such as patches for CGM, small tokens, such as key chains and, with six participants suggesting gift cards without prompting from the interviewer. When probed further, the majority of participants agreed that small prizes related to diabetes were ideal rewards. These included over-patches for continuous glucose monitoring sensors, small packets of treatment for hypoglycemia and merchandise from sponsoring companies, such as beanies and keychains.

#### 3.5.4. Live Chat Function

Participants were also asked about the concept of having a live chat function during the game or as a standalone feature of a diabetes education program. While most welcomed the idea, some disliked it, believing it would not meet their needs for authentic discussions. Additionally, one participant believed that most live chats are managed through bots, and they would prefer to speak to a real person, which would be difficult to ascertain online.

Participants had no strong preference for a peer leader or a healthcare professional managing the chat, highlighting the unique advantages of both options. Those favoring peer leaders valued the opportunity to gain real world experiences and insights about various aspects of living with diabetes. Participants' expectations of an ideal peer leader were someone older who *“knows what they're doing”*, knowledgeable, emphatic, patient, understanding, nonjudgmental and appropriately trained to manage conversations and provide credible information. On the other hand, those preferring healthcare professionals appreciate the convenience of asking clinically relevant questions without waiting for their next appointment.

*“*[*I'd prefer*] *someone who's lived with diabetes. I feel like healthcare professionals don't actually understand that*, *like a lot of things with diabetes is easier said than done” – participant 011*

*“it'd also be good to not have to schedule an appointment* [*with a healthcare professional*]*” – participant 003*

When discussing the possibility of a broader diabetes online community chat on social media platforms, all participants expressed interest in using, such a resource. However, some indicated they would prefer to observe rather than actively participating in discussions. Regarding platform preferences for a diabetes online community chat group, Instagram and Facebook emerged as the most popular choices, followed by Snapchat, Discord, WhatsApp and Reddit, in descending order of preference.

## 4. Discussion

The current study aimed to understand the experiences, perspectives, facilitators and barriers faced by adolescents with T1D in Australia and to identify approaches to inform future diabetes education programs and services. Overall, participants highly valued opportunities for diabetes education beyond the clinical setting, which provided emotional support, information in an age-appropriate format and an avenue to help family and friends better understand the diabetes lived experience. However, participants relied heavily on their parents to know about, such opportunities. Overwhelmingly, participants preferred in-person, interactive education programs. While gamification was a key attractor to participants, the desirability of a live chat function was divided.

### 4.1. Benefits of Diabetes Camps and Events

Our findings reported high engagement and satisfaction with diabetes camps and events primarily due to meeting peers with T1D and receiving diabetes-related information tailored to them. These findings align with literature highlighting that adolescent emotional wellbeing as a significant benefit to diabetes camps [14]. Opportunities for peer support are key to improving emotional wellbeing and reducing isolation, by fostering a sense of community and belonging through shared experiences and supporting empowerment in diabetes self-management [15–17]. Alongside companionship, peer support offers emotional support, instrumental support and informational support built upon an understanding of the lived experience of T1D, which cannot be obtained through family and friends without T1D [15].

The importance of support from peers with diabetes was reflected in findings from the current study where participants found it frustrating when parents expressed an understanding of the lived experience through their management of the condition on behalf of their child. However, the burden of diabetes on parents and the family associated with the responsibility of managing their child's T1D is equally valid and should be acknowledged [18]. Therefore, it is important to deliver tailored, relevant diabetes-related information to family members and friends to support adolescents with T1D. The current study highlights that diabetes camps and events benefit adolescents by educating their support networks. Previous research has suggested that for parents and adolescents, educational information could also include strategies to support and build adolescents' skills and confidence towards independent diabetes self-management [17, 19]. These topics were echoed by study adolescents whose educational topics or content suggestions reflected progression towards independent diabetes self-management.

Interestingly, the current study only had a small number of participants who reported independently seeking out diabetes-related information, including diabetes camps and relevant events, often through social media. The majority of participants relied heavily on their parents for, such information, as is often the case in this age group highlighted in the literature [20]. Social media exposes adolescents to a wider variety of diabetes online communities, which provide similar peer support opportunities around emotional, instrumental and informational support [21]. Previous evidence has found that active participation in diabetes online communities can also improve empowerment and motivation for self-management [21]. As such, it could be argued that adolescents who seek out diabetes-related information online are in a period of transition to independent self-management. However, this also emphasizes the need for diabetes-related information, including relevant events to be targeted to and accessible directly by adolescents through platforms where they gather, rather than pitched to parents and carers solely.

### 4.2. Integration of Healthcare Professionals and Peer Support

Findings from the current study highlight that healthcare professionals can play a more prominent role in diabetes education through avenues beyond traditional clinical care, such as through live chat functionality in online diabetes education and service programs, as has been reflected in previous research [21, 22]. Unfortunately, the prevalent medical model of care through outpatient clinics may have contributed to this underutilization of healthcare professionals through innovative approaches [23]. Participants emphasis on interacting with empathetic healthcare professionals with appropriate diabetes expertise could suggest these comments likely stem from previous poor experiences with healthcare professionals, which have often been reported in research, particularly among the young adult population [24–26]. These findings emphasize the importance of having an appropriately upskilled empathetic diabetes workforce to provide tailored services to specific populations, such as adolescents as they transition from pediatric to adult care and beyond [27].

The importance of a peer-led community to validate day-to-day experiences of living with diabetes aligns with previous studies [21, 22]. A key benefit of online peer support groups, as reflected in research, is the ability for passive participation, where individuals observe conversations with others without active contribution to discussions, which could trigger additional questions or commentary towards their own learning that they would not have otherwise thought of [21, 22].

Recurring themes from participants highlighted the importance of integrating peer support into routine diabetes education and care, a practice well supported by evidence [22]. The added benefit around convenience and easy accessibility to online peer support communities through social media platforms as highlighted by participants and past studies is another benefit to adolescents [21, 22]. While diabetes online communities can provide emotional support, individuals can be exposed to a wide variety of diabetes-related information, including risk-taking behaviors, such as alcohol and drug use and inappropriate use of insulin for weight manipulation [21]. Therefore, the role of healthcare professionals is critical in providing age-appropriate diabetes education, assisting adolescents to make informed-decisions and to undertake regular assessments around emotional wellbeing and healthy coping behaviors [21, 22]. In particular, as adolescents progress through major transition points in life, such as moving out of home, engagement with healthcare professionals serves as a critical opportunity to support them in conjunction with peer support services [28].

### 4.3. Importance of Codesign in Diabetes Education Programs and Services

Ultimately, as reflected in the variety of responses from participants in modality preferences for diabetes education programs and services, it is important to involve adolescents in its design to meet the needs of different local communities and age groups. Ensuring that a model of care is codesigned and developed that supports the priorities of adolescents and young adults undergoing pediatric to adult care, while being minimally disruptive to their lives, enhances clinic attendance and ongoing engagement with healthcare professionals [8]. For example, gamification was broadly accepted among participants as a feature within diabetes education programs and services. Gamification and reward systems can be powerful motivators for self-management behaviors [29, 30] However, they must be designed with an understanding of the psychological mechanisms that drive adolescent engagement to maximize efficacy [29, 30]. A key value of co-designing health programs and services with people with lived experience is the unique perspective they bring that could innovate models of care [31, 32]. This can be evidenced by suggestions provided by participants for incorporating interactive components in diabetes education programs and services, such as using an escape room format where players have to solve a series of puzzles to finish the game.

## 5. Strengths and Limitations

The current study offers valuable insights into the experiences and perspectives of adolescents with T1D when it comes to designing an ideal diabetes education program or service. A key strength lies in exploring both experiences and perspectives from attendance to diabetes camps and events as well as desired program and service features through hypothetical questioning. However, limitations associated with the sample population and recruitment strategy should be acknowledged. The sample population had a strong focus on a single metropolitan area, with access to diabetes technology, such as insulin pumps, and support from parents or carers. Such a sample population potentially reflects a higher socioeconomic demographic representation, which may not have allowed for the identification of nuances for those living in rural and remote areas. Future research with a more diverse purposive sample could draw on the challenges of those living in remote and rural areas, which may impact on their ability to access diabetes education programs and services due to geographical location. Additionally, exploration of needs and preferences from individuals within lower socioeconomic backgrounds is key, due to its association with diabetes technology uptake [33]. Specifically, within Australia, Automated Insulin Delivery (AID) systems are used by less than 20% of those living with T1D due to current funding model for insulin pumps and rising costs of private health insurance premiums [34]. Partnering with these priority populations can help address the disparities in access to diabetes care based on socioeconomic status and geographic location.

## 6. Conclusion

Overall, these findings highlight the value peer support brings to adolescents living with T1D while complementing diabetes education and care provided by healthcare professionals. Clearly, there is no one-size-fits-all approach when it comes to diabetes education and program service delivery. However, moving beyond traditional educational models and incorporating social interaction strategies across various formats (in-person, virtual, and hybrid) are likely to create a more inclusive and engaging environment to support the wellbeing of adolescents with T1D. To meet the varied diabetes education and program service needs of a diverse adolescent group with T1D, codesign approaches remain a key solution to develop tailored approaches to engage and support this population towards successful transition care, setting them up for diabetes self-management success in the future.

## Figures and Tables

**Table 1:** Topics of interest ranked by frequency mentioned.

• Managing diabetes and sports • Managing mental health and emotional wellbeing with diabetes • Managing traveling and diabetes • Moving away from family • Diabetes research updates • Broad diabetes management strategies and understanding terminologies • Diabetes technology landscape and updates • Managing alcohol with diabetes • Managing driving with diabetes

## Data Availability

The datasets generated in this study are available from the corresponding author upon reasonable request, with the permission of the University of Technology Sydney.

## Supporting Information

Supporting Information 1 Appendix 1. Interview schedule.

Supporting Information 2 Standards for Reporting Qualitative Research (SRQR) checklist.
