# Supplementary material for: The Need for Peer Support and Codesigned Services: A Qualitative Study to Understand Diabetes Education Program Needs of Adolescents With Type 1 Diabetes
Source: Pediatr Diabetes. 2025 Jun 24;2025:1843544. doi: 10.1155/pedi/1843544 (PMC12213036; doi:10.1155/pedi/1843544)
Supplement: Supporting Information 1 — Appendix 1. Interview schedule. [file 1843544.f1.docx]

## Part 1 – You and your diabetes

- Can you share with me how you are currently managing your diabetes?
  - Prompt: Insulin pump vs MDI, CGM vs SMBG
- Can you tell me about the healthcare professionals you see for your diabetes?
  - Prompt: F2F vs telehealth
- Can you tell me about any diabetes events or activities you have been to?
  - Prompt: Camps, peer support, webinars etc.
- Who do you think is ‘in charge’ of your diabetes or making diabetes-related decisions now?
  - Prompt: Parents, yourself or both
  - Prompt: Provide example i.e. After your main meals like breakfast, lunch and dinner, who often count the carbohydrates in that meal? Who decides how much bolus you will need?

## Part 2 – The needs

- Do you think there is a need for diabetes events or activities for you and other teens who live with diabetes?
  - Expand further on response: why (what is missing/ what would you like to get out the event/activity) /why not
  - Prompt: event/activity to help your friends understand more about living with diabetes?

## Part 3 – The programs or services you know

- What are some diabetes events, programs or activities you have heard of?
  - Prompts:
    - How did you hear about them?
    - Attended?
      - If yes: why/experience/cost/what you liked and disliked/what could be improved/would you go back or recommend
      - If no: why/what would have made you go (topics are not relevant, timing, travel, cost or not interested)
- Where do you get diabetes information from?
  - Prompt: social media, clinical appointment, asking others
  - Prompt: Social media: who do you follow? Why?
- Outside of diabetes, have you attended or know of any other health-related events/activities?
  - If yes, expand on program details
    - F2F/virtual
    - Content
    - Timing
    - Experience if attended

## Part 4 – Ideal program or service

- What format of diabetes events or activities would you prefer?
  - Prompt: F2F, virtual, mixed
  - Expand on why
- What timing would work best for you?
  - Prompt: School term or school holiday, weekday vs weekend vs school hours, after school
- What topics you like to know more about when it comes to living with diabetes?

## Part 5 – This or that: a hypothetical educational service

- An award system – a point system with rewards: Yes or No
  - Type of rewards?
- Games
  - Video games or physical activity games
    - Video game prompt: action games, adventure games, role-playing games, simulation games, sports games
    - Physical activity game prompt: involve running around or an activity or just being outside
- Live chat function
  - With healthcare professionals or trained peer leaders who live with diabetes?
  - What type of healthcare professional?
  - Peer leader attributes?
  - What type of questions do you think you will ask them?
- Community chat function like on social media
  - Preferred social media platform?
- Any other feature or function that you get you interested?

## Part 6 – Closing

- Anything else you like to tell us that we haven’t covered
